# The daily dose of testosterone-replacement therapy dependence from the body mass index in FtM transgender PATIENTS

**DOI:** 10.14341/probl12829

**Published:** 2022-04-30

**Authors:** L. Y. Sergeeva, A. Yu. Babenko

**Affiliations:** Almazov National Medical Research Centre; Almazov National Medical Research Centre

**Keywords:** hormone replacement therapy, hormonal correction of transgender patients, body mass index

## Abstract

**BACKGROUND:**

BACKGROUND: The basis for the management of transgender patients is the use of various hormonal correction schemes necessary for changing the hormonal sex and, possibly, further preparation for surgical correction. Currently, the choice of the starting dose and the scheme is carried out empirically, which lengthens the period of selection of therapy and increases the risk of its complications. Taking into account the individual characteristics of the patient can help in optimizing therapy.

**AIM:**

AIM: Investigate Factors Affecting the Daily Demand for Testosterone Ester Blends in Transgender Men

**MATERIALS AND METHODS:**

MATERIALS AND METHODS: This study is a case-control observational study. Patients included prior to initiation of testosterone replacement therapy. The analysis of factors interrelated with the daily requirement of testosterone preparations was carried out. Among the factors of interest, the body mass index (BMI), the results of blood tests for total testosterone and the functional state of the liver and kidneys are considered. Testosterone replacement therapy (TRT) regimens were evaluated in transgender men. For the calculation, we used the formulas for BMI and the average daily dose of testosterone. Based on the data obtained, conclusions were drawn that allow determining the necessary TRT scheme in different trans-gender men at an early stage of hormonal correction.

**RESULTS:**

RESULTS: Our study included 58 transgender FtM patients who were prescribed testosterone preparations with an identical composition. We found a positive correlation between BMI and testosterone dose in patients of group II (p = 0.04).

**CONCLUSION:**

CONCLUSION: In the conclusion, the obtained schemes of hormonal sex reassignment with a minimum risk of possible complications are presented. Our results demonstrated a relationship between BMI in overweight and obese patients and the need for TRT. For patients with a BMI of 25 to 29 kg / m2, the interval between injections of a mixture of testosterone esters does not differ significantly from that in the group with a BMI below 25 kg / m2 and averages once every 18 days, and in the group with a BMI ≥ 30 kg / m2 tested testosterone ester preparations should be prescribed once every 2 weeks (14 days).

## Abstract (ru)

**ОБОСНОВАНИЕ:**

ОБОСНОВАНИЕ. Основой ведения трансгендерных пациентов является использование различных схем гормональной коррекции, необходимых для трансгендерного перехода и, возможно, дальнейшей подготовки к хирургической коррекции. В настоящее время выбор стартовой дозы и схемы осуществляется эмпирически, что удлиняет период подбора терапии и повышает риск ее осложнений. Учет индивидуальных особенностей пациента может помочь в оптимизации терапии.

**ЦЕЛЬ:**

ЦЕЛЬ. Исследовать факторы, влияющие на суточную потребность в смесях эфиров тестостерона у трансгендерных мужчин.

**МАТЕРИАЛЫ И МЕТОДЫ:**

МАТЕРИАЛЫ И МЕТОДЫ. Данное исследование является наблюдательным «случай-контроль». Включались пациенты до начала тестостерон-заместительной терапии (ТЗТ). Выполнен анализ факторов, взаимосвязанных с суточной потребностью препаратов тестостерона. Из интересующих факторов рассмотрены индекс массы тела (ИМТ), результаты анализов крови на общий тестостерон и функциональное состояние печени и почек. Проведена оценка схем ТЗТ у трансгендерных мужчин. Для расчета использованы формулы ИМТ и среднесуточной дозы тестостерона. На основе полученных данных сделаны выводы, позволяющие определить необходимую схему ТЗТ у разных трансгендерных мужчин на раннем этапе гормональной коррекции.

**РЕЗУЛЬТАТЫ:**

РЕЗУЛЬТАТЫ. В наше исследование были включены 58 трансгендерных пациентов FtM, которым назначались препараты тестостерона с идентичным составом. Нами была установлена положительная корреляция между ИМТ и дозой тестостерона у пациентов II группы (р=0,04).

**ЗАКЛЮЧЕНИЕ:**

ЗАКЛЮЧЕНИЕ. В заключение приведены полученные схемы гормональной коррекции трансгендерного перехода с минимальным риском возможных осложнений. Наши результаты продемонстрировали наличие зависимости между ИМТ пациентов с избыточной массой тела и ожирением и потребностью в ТЗТ. Для пациентов с ИМТ от 25 до 29 кг/м2 интервал между инъекциями смеси эфиров тестостерона не отличается существенно от такового в группе с ИМТ ниже 25 кг/м2 и составляет в среднем 1 раз в 18 дней, а в группе с ИМТ≥30 кг/м2 изученные препараты эфиров тестостерона необходимо назначать 1 раз в 2 нед (14 дней).

## RELEVANCE

At present, gender dysphoria is identified in approximately 25,000,000 of adults globally (about 0.5% of world population). Such individuals all the more frequently seek medical assistance, which signals a high relevance of the problem. Among the patients with this syndrome, 80% are FtM individuals, i.e., biological females that identify themselves as males [1][2].

Hormonal correction for FtM transgender patients is conducted with androgens drugs. Individuals seeking such medical assistance vary in their age and anthropometrics. Therefore, more personalised management protocols have to be developed for this group of patients, depending on their medical history data, physical characteristics and other data, including concomitant diseases and age [3]. There is currently no consensus as to which factors contribute to efficiency of a hormonal correction scheme and what exactly drives changes in testosterone level and physical characteristics so as to make an individual approximate the opposite sex. In Russia, testosterone replacement therapy is most often carries out using testosterone ethers (Omnadren, Sustanon, or Nebido). Sustanon and Omnadren, which were used in this study, have identical content (a mix of four testosterone ethers: testosteroni propionatis, phenilpropionatis, isocapronatis, and decanoatis) and identical pharmacodynamics: the peak effect comes in 7 to 10 days, and the effect ends 14 to 21 days following the injection. According to recommendations of the European Society of Endocrinology [4], the therapy should raise testosterone level to the target range of male physiological norm (400 to 700 ng/dL).

## OBJECTIVE

Examine the factors affecting daily requirement of testosterone ethers mix in transgender males.

## MATERIALS AND METHODS

## Study venue and time

Venue. St. Petersburg city budgetary institution of health care, City out-patient clinic № 3, department № 2.

Time. The study was conducted between September 2020 and February 2021.

## Examined populations

One population was examined: FtM transgender patients.

Criteria of inclusion:

Criteria of exclusion:

## Method of sampling the examined population(s)

Every member of the relevant population was included in the study

## Research design

Through the last two years, 80 FtM transgender individuals registered in the out-patient clinic № 3; 58 of them were included in this study as they had started receiving therapy with Omnadren or Sustanon. The remaining 22 patients have been using Nebido or a transdermal drug (Androgel). This study is an observational one (a case-control study), a dynamic and prolonged one (patients were observed for six months).

At the start of treatment, all patients were recommended the same interval: once every three weeks ±2 days if necessary. After the second injection, the patients’ testosterone levels were measured exactly between the injections and immediately prior to the next injection. Based on laboratory data, injection intervals were adjusted so as to keep testosterone level within the target range. Then, after two more injections made with the new interval, we measured the testosterone levels again and found that some patients had reached their target values (including those who had reached their target values already with initially recommended intervals) whereas some had not.

As potential factors affecting the patients’ daily requirement of testosterone, we evaluated their age at the start of testosterone replacement therapy, BMI, initial testosterone level and presence of liver or kidney diseases.

## Methods

In this study, we used formulas to calculate a daily testosterone dose and to calculate BMI; we also used scales and height metre.

General testosterone level was measured through electrochemiluminescence immunoassay (ECLIA); reference interval: 8.64–29 nmol/L.

Daily testosterone dose calculation:

Drug dosage in 1.0 mL 250.0 mg divided by the number of days, as per each patient’s individual testosterone replacement therapy scheme.

BMI calculation: body weight (kg) divided by squared height (m)

## Statistical analysis

The tables below present our findings as median, lower-quartile and upper-quartile values. The two groups of patients examined in this study are divided by their BMI and unrelated to one another. Therefore, to verify the statistical significance of group medians, Mann–Whitney test was applied. We also used Kruskall-Wallis test to establish differences between the medians for all four subgroups. In order to evaluate the correlation between BMI and daily dose of testosterone, Spearman correlation analysis was applied to two groups.

## Ethical review

Ethical review was conducted by Ethical Board of Almazov National Medical Research Centre, St. Petersburg, Russia. Verdict: No conflict of interest found. Resolution: Approve the study (Record no. 21.10.21 signed on 04 October 2021).

## FINDINGS

According to criteria of inclusion, 58 FtM patients were included in this study. Main properties of these patients prior to testosterone replacement therapy start are provided in Table 1.

**Table:** Table 1. Main properties of patients included in this study Note: BMI – body mass index; ALT – alanine transaminase; AST – aspartate transaminase

Indicator	median (lower-quartile; upper-quartile) values
Age (years)	21.50 (20.00; 26.00)
Initial testosterone level (nmol/L)	1.97 (1.68; 2.24)
BMI (kg/m2)	21.90 (19.00; 26.20)
ALT (IU/L)	20.45 (19.00; 22.00)
AST (IU/L)	20.40 (19.00;24.00)
Total bilirubin (μmol/L)	11.55 (10.30;14.60)
Glucose (mmol/L)	4.40 (4.00; 5.00)
Total cholesterol (mmol/L)	4.00 (3.50; 4.30)
Triglycerids (mmol/L)	1.00 (0.98; 1.10)
Creatinine (μmol/L)	64.00 (58.00; 70.00)
Glomerular filtration rate (mL/min/1.73 m2)	134.00 (126.00;141.00)

No significant liver or kidney pathology was found in any of the patients included in this study, nor there was any correlation between the parameters defining their condition and their daily testosterone doses. The patients were divided in two groups depending on their BMI.

Group I: patients without overweight

Subgroup 1: those with BMI deficiency (BMI under 18.5), n = 20 (34%)

Subgroup 2: those with normal BMI (BMI from 19 to 24, both inclusive), n = 18 (31%)

Group II: overweight patients

Subgroup 3: those with overweight (BMI from 25 to 29), n = 14 (24%)

Subgroup 4: those with obesity (BMI from 30 to 35) n = 6 (11%)

Thus, 65% of patients had BMI deficiency or normal BMI, whereas 35% of patients had overweight or Degree 1 obesity.

All patients were prescribed a starting therapy with 1 mL of Omnadren or Sustanon 250 mg once every three weeks; thus, daily testosterone dose was similar for all subgroups: median 13.15 [ 11.9; 17.8], р=0.76 (Mann-Whitney-Wilcoxon criterion) (Figure 1).

**Figure:**
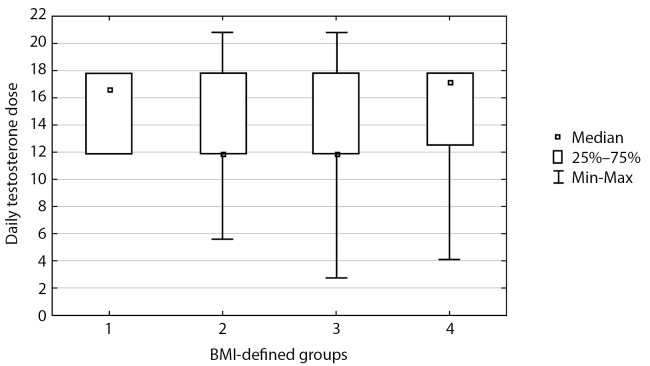
Figure 1. Daily dose of testosterone ethers

Calculations of daily testosterone dose were made for each patient based on their personalised testosterone replacement therapy scheme.

After two injections following therapy start, total blood testosterone was measured in all patients both at peak time of testosterone ethers effect and prior to the next injection. It was found that during testosterone replacement therapy median testosterone level measured exactly between the injections in all patients was 33.00 nmol/L [ 27.20; 39.40], whereas median testosterone level measured prior to the next injection in all patients was 18.95 nmol/L [ 16.50; 23.90]. Median daily dose of testosterone ethers for all patients was 14.88 [ 11.90; 17.80].

Then, three months following testosterone replacement therapy start, blood serum testosterone was measured in the two groups of patients, both at peak time and at “dropdown” time immediately prior to the next injection. The findings are presented in Table 2.

**Table:** Table 2. Total testosterone at peak time and immediately prior to the next injection for both groups

BMI	Total testosterone (nmol/L) at peak time, median (Q1; Q3)	Total testosterone (nmol/L) prior to the next injection, median (Q1; Q3)
Under 25 kg/m2	38.0 [ 30.0; 39.75]	22.20 [ 18.55; 26.90]
At or over 25 kg/m2	25.85 [ 23.8; 29.3]	16.3 [ 13.5; 18.0]
p	0.0002	<0.0001

The table shows that these groups displayed a substantial difference in testosterone level both at peak time (p=0.0002) and prior to the next injection (p<0.0001), and testosterone level was substantially higher in patients with normal body weight. These differences are illustrated in Figures 2 and 3. In Group I, at peak time of testosterone ethers effect, an elevated testosterone level was observed which exceeded the upper limit of male physiological norm; therefore, a correction of testosterone replacement therapy scheme was performed for this group of patients. Thus, required daily dose of testosterone drugs is substantially different for Subgroup 2 in Group I. These patients will require a correction of testosterone replacement therapy scheme more often than those in Group II. As a result, it took about two months to select a suitable injection interval enabling to maintain Group II patients’ testosterone within the target level and 3–4 months to do so for Group I.

**Figure:**
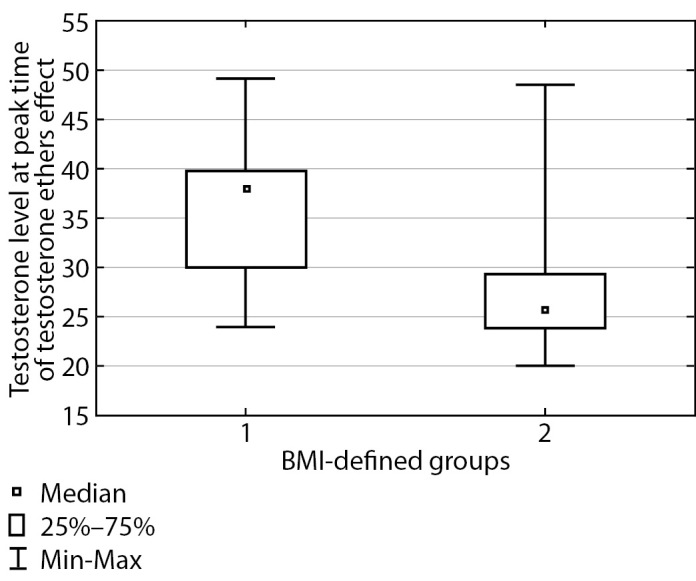
Figure 2. Total blood testosterone at peak time of testosterone ethers effect

**Figure:**
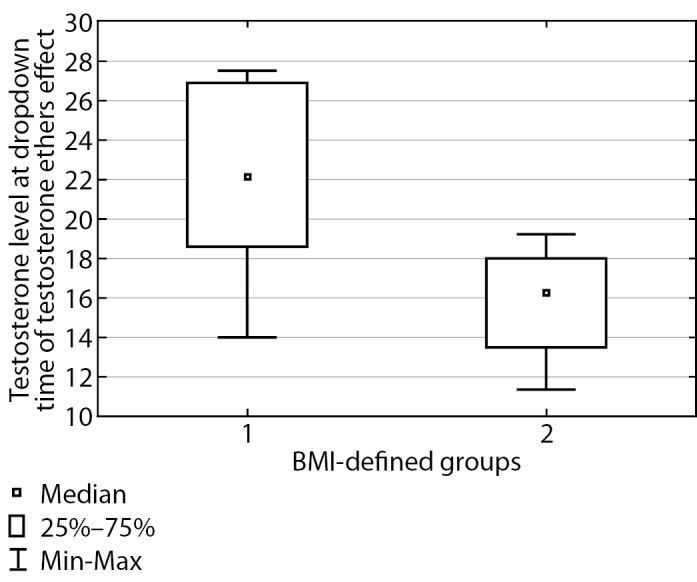
Figure 3. Total blood testosterone immediately prior to the next injection

In order to determine the correlation between BMI and daily dose of testosterone ether drugs, Spearman correlation analysis was conducted. No correlation between BMI and daily dose of testosterone was found in Group I. In Subgroup 4 of Group II, a significant moderate positive Spearman correlation was identified in patients with obesity: Rs=0.46, р=0.04 (Figure 4).

**Figure:**
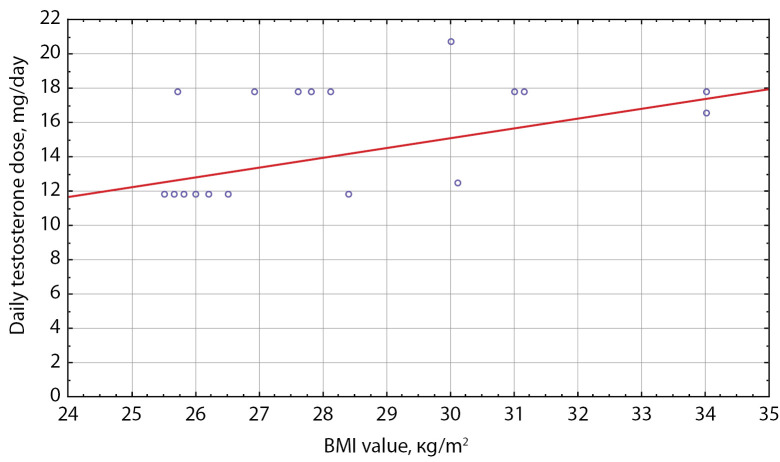
Figure 4. Daily testosterone dose in Group II patients

In order to determine the daily dose with reference to BMI, which would enable arriving at a suitable treatment scheme faster, patients in each group were divided into two subgroups: those with testosterone level within the target range at both control points and those with testosterone level outside the target range; average daily dose was determined in both these subgroups.

In Group I, average daily dose of testosterone replacement drugs did not substantially differ between the subgroups, and median injection interval was 18 days. In Group II, median injection interval was 14 days, while testosterone level was substantially lower than in Group I patients.

Testosterone level in Group II patients was substantially lower than in those of Group I. Moreover, for Group I, daily dose of testosterone replacement drugs was 17.2 mg in the subgroup with target testosterone levels (with injection intervals at 14–15 days) and 12.53 mg in the subgroup with excessive testosterone levels at mid-injection interval (with injection intervals at 19–20 days).

## DISCUSSION

## Sample representativeness

It is impossible to evaluate sample representativeness with reference to total population, since the sample was made up of all patients who turned up at this out-patient clinic № 3 only.

## Mapping against other published studies

Overweight and obesity in FtM transgender patients require special attention since adiposis, especially visceral fat amassing, not only carries a risk of atherosclerosis and insulin resistance, which in turn amplifies the risks associated in this cohort with developing hyperandrogenism in genetic females [5], but also poses additional difficulties for hormonal correction in transgender patients. The more adipose tissue is present, the more active aromatase becomes, thus enhancing testosterone transformation into estradiol [6]. This may explain the correlation which we have identified between BMI and blood serum testosterone in patients receiving the same doses (schemes) of testosterone replacement therapy. This factor should be taken into account when managing transgender patients, especially those with overweight and obesity.

To date, a large number of studies have been published that indicate a correlation between testosterone deficiency and high BMI values [7][8]; however, we have not been able to find any study that describes BMI impact on daily required dose of testosterone replacement drugs. Given that many other hormones (such as thyroxine or insulin) are prescribed with reference to the patient’s body weight and further given that the growth of adipose tissue increases the activity of aromatase which enhances testosterone transformation into estradiol, it appears reasonable to suppose a correlation between BMI and daily required dose of testosterone.

## Limitations of this study

The limitations of this study are related to the problem of sample representativeness with reference to total population (as the sample was made up of patients who turned up at St. Petersburg Public GP Surgery 3, Outpatient Department 2 only) and relatively small sample size.

## Need for further research

It is necessary to investigate additional factors affecting the daily required dose of testosterone in patients with BMI deficiency, with normal BMI and with obesity. At present, we may only suggest that this daily required dose depends on the activity of aromatase; therefore, further studies are needed to assess the activity of aromatase and gain more understanding of the mechanisms accounting for the differences we have identified. AR CAGn polymorphism is another important factor that may contribute to overweight [9] and lead to a higher daily required dose of testosterone replacement drugs [10][11].

## CONCLUSION

Our findings demonstrate a correlation between BMI in overweight and obesity patients and their daily required dose of dose of drugs for TRT. For patients with BMI between 25 and 29 kg/m2, the interval between injections of testosterone ethers mix did not substantially differ from that in the group with BMI under 25 kg/m2 and amounted to an average of 18 days, whereas for the group with BMI at or over 25 kg/m2 the testosterone drugs we examined need to be administered once every 14 days to achieve target testosterone levels.

Thus we have established a correlation between BMI and average daily required dose of testosterone in overweight and obesity patients: the higher the BMI value, the greater the daily dose of testosterone required to achieve target testosterone levels.

## ADDITIONAL INFORMATION

Funding source. This study was conducted on the authors’ own accord. No funding was raised.

Conflict of interest. The authors hereby declare no actual or potential conflict of interest related to this publication.

Authors’ contribution. Lyudmila Y. Sergeeva: criteria 1 and 2 — personal and main contribution to the drafting of the entire manuscript, research design and concept, data acquisition and analysis, interpretation of findings, tables, figures and diagrams creation; criterion 3 — finalising the manuscript; criterion 4 — willingness to accept responsibility for all aspects of this study. Alina Yu. Babenko: criterion 1 — substantial contribution to research design and concept; criterion 2 — substantial improvements of the manuscript thus increasing its scientific value; criterion 3 — approval of the final version of the manuscript; criterion 4 — willingness to accept responsibility for all aspects of this study, which implies due investigation and resolution of any issue related to the accuracy or integrity of any part thereof.
